# 1390. Assessing the Relationship between Community Social Vulnerability and Antibiotic Use in Nursing Homes

**DOI:** 10.1093/ofid/ofac492.1219

**Published:** 2022-12-15

**Authors:** Casey M Zipfel, Prabasaj Paul, Rachel Slayton

**Affiliations:** Centers for Disease Control and Prevention, Decatur, Georgia; Centers for Disease Control and Prevention, Decatur, Georgia; U.S. Centers for Disease Control and Prevention, Atlanta, Georgia

## Abstract

**Background:**

Health inequities impact healthcare delivery and outcomes, but the impacts of health inequities on antibiotic use and AR infections have been less frequently studied in nursing home residents. Nursing home residents are typically older, often have increased care needs requiring frequent contact with staff, and often have comorbidities or in-dwelling devices that make them vulnerable to infection. The community served by a nursing home may play a role in frequency and variability in antibiotic use, as health inequities may impact healthcare delivery and health status of patients, since both patients and caregivers are likely to be drawn from the surrounding community. We hypothesize that antibiotic use in nursing homes is impacted by the social vulnerability of the surrounding community.

**Methods:**

We collected all reports of the proportion of residents who received antibiotics in the previous 7 days in 2019, which are reported monthly to the CMS Minimum Data Set. We assessed the proportion of facility residents using antibiotics by social vulnerability measures (from CDC Social Vulnerability Index) of the surrounding healthcare-seeking community (Hospital Service Areas from Dartmouth Health Atlas), using a hierarchical model to control for facility factors.

**Results:**

We find considerable variability in antibiotic use across 14,908 US nursing homes (Figure 1A). After controlling for facility characteristics, facilities located in areas of greater community-level social vulnerability were associated with higher antibiotic use (Figure 1B).

**Figure:**
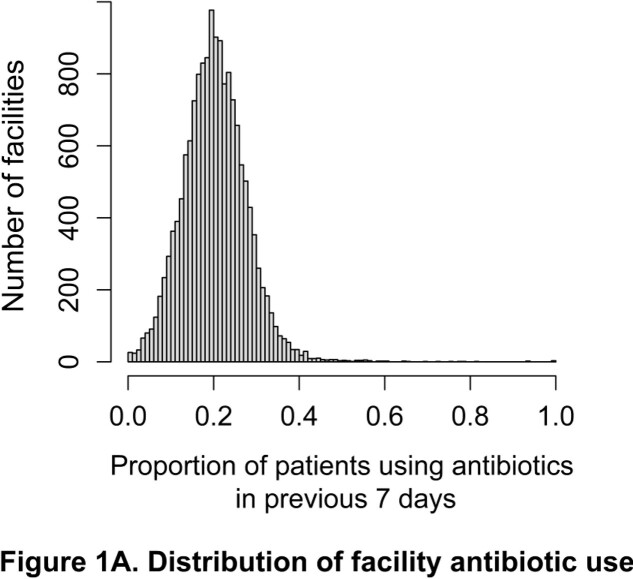

Figure 1. Antibiotic use in nursing homes and relationships with community social vulnerability. A) The distribution of the proportion of patients using antibiotics within the previous 7 days by facility.

**Figure:**
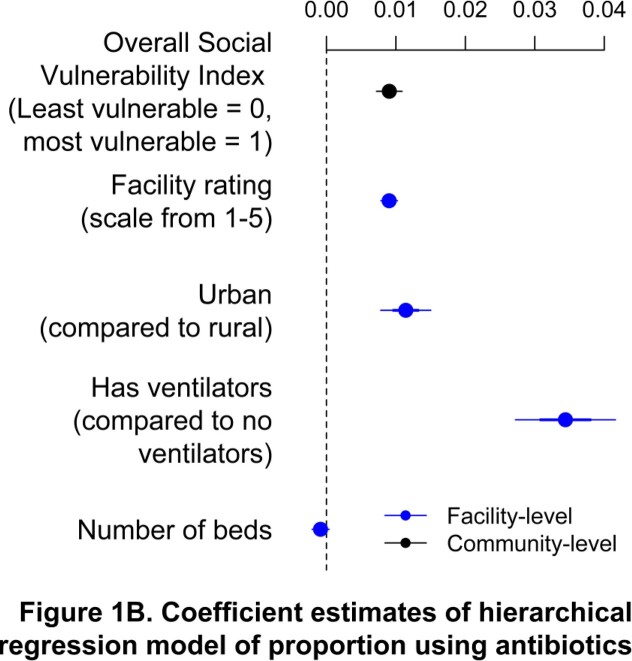

B) Coefficient estimates of a multilevel regression model. Controlling for facility-level factors (blue), community-level social vulnerability index appears to be related with more antibiotic use.

**Conclusion:**

These findings indicate that community social vulnerability is associated with healthcare delivery within facilities. This association may result from differences in antibiotic prescribing behavior, infection prevention practices, or from disparity-driven differences in the health status of the resident population. This work is important for informing epidemiological models within healthcare facilities: failing to include heterogeneity of the surrounding community could bias transmission models and decrease the accuracy of targeted public health interventions to vulnerable facilities and communities.

**Disclosures:**

**All Authors**: No reported disclosures.

